# Microsaccades, Drifts, Hopf Bundle and Neurogeometry

**DOI:** 10.3390/jimaging8030076

**Published:** 2022-03-17

**Authors:** Dmitri Alekseevsky

**Affiliations:** 1 Institute for Information Transmission Problems, B. Karetnuj per., 19, 127051 Moscow, Russia; dalekseevsky@iitp.ru; Tel.: +7-916-529-8349; 2 Faculty of Science, University of Hradec Králové, Rokitanského 62, 500 03 Hradec Králové, Czech Republic

**Keywords:** Donders’ and Listing’s law, quaternions, Hopf bundle, fixation eyes movements, drift, microsaccades, remapping, shift of receptive fields, neurogeometry, 87.19.La, 42.66.Ct, 92B20

## Abstract

The first part of the paper contains a short review of the image processing in early vision is static, when the eyes and the stimulus are stable, and in dynamics, when the eyes participate in fixation eye movements. In the second part, we give an interpretation of Donders’ and Listing’s law in terms of the Hopf fibration of the 3-sphere over the 2-sphere. In particular, it is shown that the configuration space of the eye ball (when the head is fixed) is the 2-dimensional hemisphere $S_{L}^{+}$, called Listing hemisphere, and saccades are described as geodesic segments of $S_{L}^{+}$ with respect to the standard round metric. We study fixation eye movements (drift and microsaccades) in terms of this model and discuss the role of fixation eye movements in vision. A model of fixation eye movements is proposed that gives an explanation of presaccadic shift of receptive fields.

## 1. Introduction

The main task of the visual system is processing and decoding visual information, recorded by the retinal photoreceptors, and constructing a model of the external world. The photoreceptors convert the light signal into electric signals which are sent to retinal ganglion cells and then by a conformal retinotopic mapping to LGN, then to the V1 cortex, V2 cortex etc. The visual system has a hierarchical structure and consists of many subsystems connected by direct and feedback.

The neurogeometry of vision deals with the construction of continuous models of various visual subsystems in terms of differential geometry and differential equations.

There are three level of the models of the visual subsystems:

- Static, without taking into account time, i.e., under assumption that the eye and the perceived object (stimulus) are stationary;
- Semi-dynamic, when the stimulus is stationary and the eye is moving;
- Dynamic, when both the eye and the stimulus are in motion.

Over the past two decades, great progress has been made in understanding the functional architecture of early vision in static and constructing the neurogeometric models of early vision systems (primary visual cortex V1, hypercolumns), see [1,2,3,4,5,6,7,8,9]. The models are based mostly on the results obtained in experiments on anesthetized animals.

In natural vision, the eye always participates in different movements. According to the classical experiments of A. Yarbus [10], the compensation of the eye movement leads to the loss of vision of stationary objects in 2–3 s. Moving objects remain visible, albeit poorly. Later experiments show that the most important phase of the fixation eye movements is the drift. Compensation of microsaccades does not lead to loss of vision.

It was remarked by M. Rucci, E. Ahissar and D. Burr [11].

“As there are no stationary retinal signals during natural vision, motion processing is the fundamental, basic operating mode of human vision.”

They also note that due to this there is no big difference between semi-dynamic and dynamic vision.

In the first part of the paper, we will briefly discuss the main results concerning the static vision, which are the base points to deal with dynamic one. Currently, there are some advances in the study of the dynamic case, [12,13,14,15] although the description of the visual processes becomes significantly more complicated and new phenomena arise, such as saccade remapping [16,17], shift of the receptive field, compression of the space and time during saccades [18,19]. The main difference between static and dynamic vision is the following. As it is generally accepted, in static vision all information comes from the activation of retinal photoreceptors. In dynamic vision, the process of perception is determined by the interaction of the visual information from the retina and the dynamical information about eye movements, coded in the ocular motor system.

Even when the gaze is focused on a stationary point, it participates in different type of movements, called fixational eye movements (FEM). For a long time, most neurophysiologists did not pay serious attention to FEM. The situation has changed in the last two decades, see [20]. Both experimental and theoretical works have appeared that substantiate the important role of FEM in vision. Primarily the works by M. Rucci and their coauthors [11,21,22,23,24,25] contain detailed and critical analysis on many experimental results about different types of FEM—tremor, drift and microsaccades, and new ideas about their role in vision.

In the dynamic case, the eye movements are controlled by ocular motor system and a copy of motor command, called **corollary discharge or efference copy**, is sent from superior colliculus through MD thalamus to frontal cortex. It plays an important role in visual stability, i.e., the compensation of the shift of retinal stimuli and perception stable object as stable, see [26,27,28] for results and discussions on the problem of visual stability.

A deeper understanding of the mechanism of FEM depends on further progress in description of image processing in retina, visual cortex and in ocular motor control of eyes movements.

Fixational eye movements are stochastic in nature. There were proposed various stochastic models of FEM as a random walk, see [29,30,31]. We especially note the works [32,33]. In the most works, FEM are modeled by a random walk on the plane or on a lattice in the plane. However, the information about eye rotation, which is contained in corollary discharge, treats the eye as a ball and not as a plane. For more realistic model of FEM, which will be consistent with corollary discharge information, we need more sophisticated model of saccades and drift, where such movements are considered as rotations of the eye ball. Due to this, it is important to describe the configuration space of the eye.

A priori the configuration space of eye ball $B^{3}$, rotating around its center *O*, is the orthogonal group SO(3) (which can be thought as the 3-sphere with identified antipodal points, $SO\left(3\right)=S^{3}/Z_{2}$).

A big surprise even for the great physicist and physiologist H. von Helmholtz was the law, discovered in the middle of the 19th century by F.C. Donders and supplemented by J.B. Listing. It states that, when the head is fixed, the real configuration space of eye positions is two-dimensional. More precisely, the direction of the gaze $e_{1}$ uniquely determines the position of the eye, described by the retinotopic orthonormal frame $\left(e_{1},e_{2},e_{3}\right)$. From the point of view of the modern control theory, such a constraint is quite reasonable. The difference between the motion control on the 3-sphere and on a surface is similar to the difference in piloting a plane and driving a car.

One of the main results of the work consists of interpreting Listing’s law in terms of a section $s:{\tilde{S}}^{2}\to S_{L}^{+}\subset S^{3}$ (which we call Listing’s section) of the Hopf bundles $\chi :S^{3}\to S^{2}$ over a punctured sphere ${\tilde{S}}^{2}=S^{2}\setminus \left\{-i\right\}$ where *i* is the direction to the nodal point of the eye sphere $S^{2}$ (in the standard position) and −i is the direction to the center of the fovea. Listing’s section is an open 2-dimensional hemisphere $S_{L}^{+}$ of a 3-dimensional sphere $S^{3}$, identified with the group $H_{1}$ of unit quaternions. This simple description of Listing’s law provides a way for construction of more realistic stochastic models of FEM and oculomotor system that control eyes movements. For example, denote by $S_{E}^{2}=\partial B^{3}$ the eye sphere in the standard position. Let $A,B\in S_{E}^{2}$ be two points and a=s(A),b=s(B) the corresponding points of Listing’s hemisphere $S_{L}^{+}$. Then the saccade with the initial gaze direction *A* and the final gaze direction *B* is the segment $ab\subset S_{L}^{+}$ of the unique geodesic $\gamma_{a,b}$ (the great semicircle) of Listing hemisphere $S_{L}^{+}$ (with the standard metric) through points a,b. The corresponding evolution of the gaze is the segment AB=χ(ab) of the circle $S_{A,B}^{1}\subset {\tilde{S}}_{E}^{2}\cap \Pi \left(A,B,-i\right)$ (with the deleted point −i), which is the section of the punctured sphere ${\tilde{S}}_{E}^{2}$ by the plane, generated by the points A,B,−i. So the space of saccades is the direct product $S_{L}^{+}\times S_{L}^{+}$ of two copies of Listing’s hemisphere.

We propose a deterministic model of fixation eye movements (drift and microsdaccades) in terms of Listing’s hemisphere. The microsaccades are considered as a mechanism of remapping the visual information, which depends of the choice of the salient point as the next gaze target. It gives a simple description of the presaccadic shift of receptive fields. We use this model to define a distance between point stimuli A,B. Then we shortly recall the basic fact of diffusion geometry, initiated by R.R. Coifman and S. Lafon [34,35], and discuss the extension of the model to the stochastic case, when the drift is considered as a random walk on Listing’s hemisphere, in the framework of diffusion geometry.

## 2. Information Processing in Early Vision in Static and Functional Structure of Retina and Primary Visual Cortex

In static, visual information is coded in firing of retinal photoreceptors, cones and rods. In the first approximation, the input function of the retina may be considered as the function I(x,y) on retina, which describes the density of energy of light, recorded by photoreceptors. The visual information is primary processing in retina and it sent to primary visual cortex V1 and then to V2, V3 and other visual systems for further processing and decoding. The visual information is coded in visual neurons which are working as filters that is functionals on the space of input function, which value depends only on the restriction of the input function to a small domain D⊂R of the retina, called **receptive filed (RF)**. The linear neurons are working as linear filters, i.e., the linear functionals, described as the integral $\int_{D}f\left(x,y\right)W\left(x,y\right)d\mathrm{vol}\;$ of the input function with some weight W(x,y), called the **receptive profile**. In reality, most visual neurons have spatiotemporal character, that is their reply depend also on time integration of the input function.

### 2.1. The Eye as an Optical Device and Input Function

**Figure 1:**
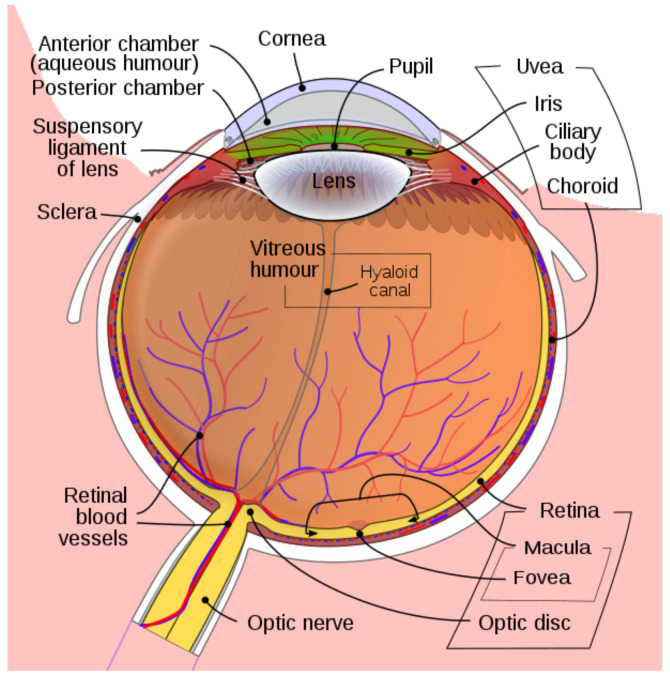
The Human Eye. Adapted from Wikipedia.

The eye is a transparent ball $B^{3}$ together with a lens *L* which focuses light rays to the retina *R*, see Figure 1. The retina occupies a big part of the boundary sphere $S^{2}=\partial B^{2}$ of the eye ball. The lens is formed by the cornea and the eye crystal. We will assume that the optical center of the lens or nodal point *N* belongs to the eye sphere $S^{2}$.

**Figure 2:**
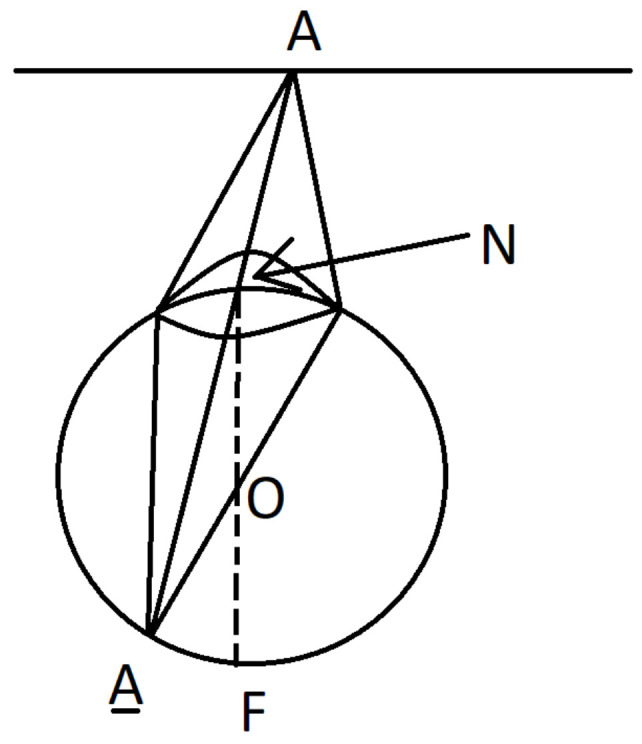
Central projection.

A beam of light emitted from a point *A* of a surface Σ and passing through the nodal point *N* is not refracted and falls to the point $\bar{A}=\ell_{AN}\cap R$ of the intersection of the retina *R* with the ray $\ell_{AN}$. A beam from the point *A* which passes through any other point of the lens is focused and come to the same point $\bar{A}\in R$. So we get a **central projection of the surface Σ to retina *R* with center *N*** given by the map

$$\pi :M∋A\to \bar{A}=\ell_{AN}\cap R\subset R,$$

where $\bar{A}=\ell_{AN}\cap R$ is the second point of intersection of the ray $\ell_{AN}$ with the retina *R*, see Figure 2. The central projection generically is a local diffeomorphism.

Note that if M=Π is the frontal plane (orthogonal to the line of sight) which is far enough away compared to the size of the eyeball, then the central projection $\pi :\Pi \to R\subset S^{2}$ is approximately a **conformal map**.

The (density of) energy of light $I_{R}\left(\bar{A}\right)$ coming from a point A∈Σ of the surface to the point $\pi \left(A\right)=\bar{A}$ of retina is approximately proportional to the (density of) energy of light I(A), emitted from the point *A*. So the input function

$$I_{R}:R\to R^{\geq 0},\;\;\;\bar{A}\to I_{R}\left(\bar{A}\right)$$

of the retina (where $R^{\geq 0}$ is the set of non negative numbers), contains information about the density I(A) of energy of light, emitted from the surface Σ. The aim of the static monochromatic vision is to extract from the input function $I_{R}$ information about geometry of the surface. We will not speak about other characteristics of the recorded light, for example, the spectral properties, which are responsible for color vision. It seems that the polarisation plays no role in human vision.

It was discovered by D. Hubel and T. Wiesel, that the most important characteristic of the detected stimulus are the contours, i.e., the level sets of the input function $I_{R}\left(x,y\right)$ with large gradient. J. Petitot [5] gave a precise geometrical formulation of this claim as a statement that simple neurons of V1 cortex detect infinitesimal contours, i.e., 1-jets of contours, considered as non parametrized curves. One of the main task of the higher order visual subsystems is to integrate such infinitesimal contours to global ones.

### 2.2. Retina

#### 2.2.1. Anatomy of Retina

**Figure 3:**
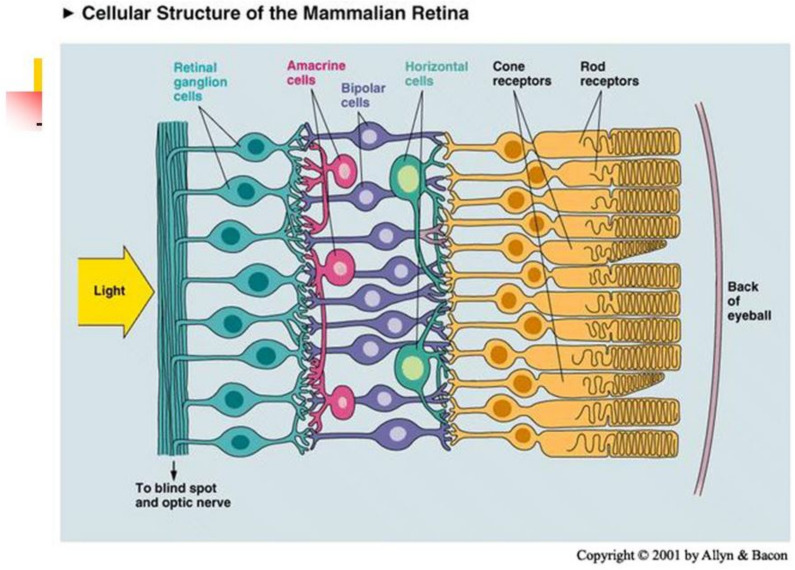
Anatomy of retina.

**Retina** consists of 5 layers. In human there are in approx. 80 different types of cells. The bottom layer consists of receptors, photoelements which transform light energy into electric signals, see Figure 3. They measure the input function

$$I_{R}:R\to R^{\geq 0}$$

and send information to ganglion cells. In fovea, one cone is connected with 1 ganglion. In periphery, one rode is connected with $10^{2}$–$10^{3}$ ganglions. There are 1 million of ganglions and 125–150 millions of receptors.

#### 2.2.2. Ganglion Cells as Marr Filters

**Figure 4:**
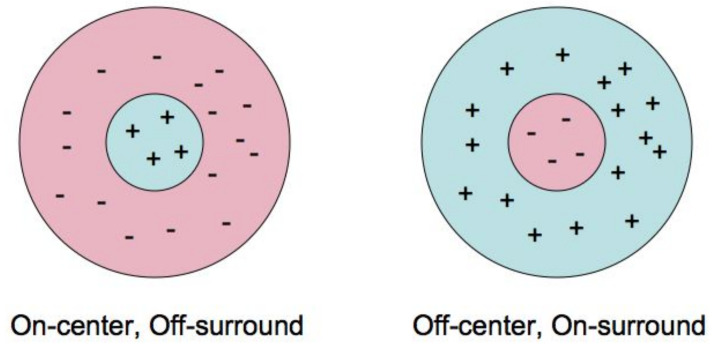
On and Off Kuffler cells.

**Figure 5:**
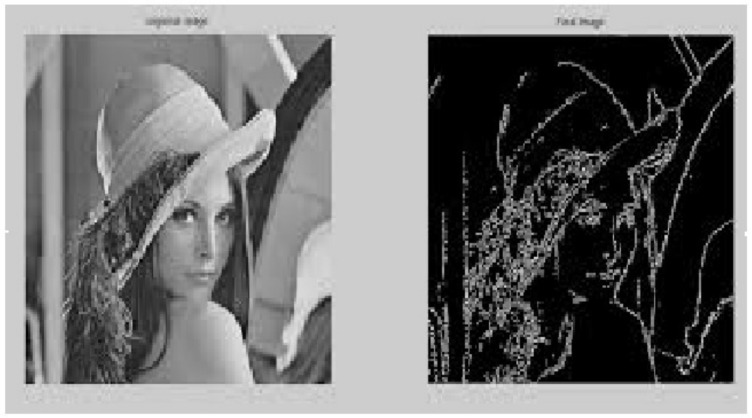
Action of Marr filter.

It was discovered by S. Kuffler that the receptive field of a typical ganglion cell is rotationally invariant (isotropic) and contains central disc and surround ring. It is working as a linear filter with receptive profile which is ether positive in the central disc and negative in the ring or vice versa. In the first case, Kuffler called it ON-cell and in the second one OFF-cell, see Figure 4. D. Marr showed that the filter with Laplacian of the Gauss function as the receptive profile gives a good model of Kuffler cell and proved that image processing by a system of such filters turns a picture into a graphic image, see Figure 5. The purpose of the information processing in retina is to regularize the input function, eliminate the small artifacts of the retina image and to highlight the contours, which are the main objects of perception in early vision.

#### 2.2.3. Information Processing in Retina. Two Pathways from Receptors to Ganglion Cells

There are two pathways from receptors to ganglion cells: **Direct path: receptor–bipolar–ganglion** activates the center of ganglion cells, which work as a linear filter. Antagonistic surround is activated by (linear) negative feed back from horizontal cells **via indirect path: receptor–horizontal cell–(amacrine)–bipolar–ganglion.** A nonlinear rectifying mechanism (associated with contrast gain control) is related with amacrine cells.

For sufficiently small contrast, ganglion P-cells is working as linear Marr filter. *M*-cells, responsible for perception of moving objects, are working as essentially non-linear filters. Response depends on stimulus contrast and temporal frequency [36].

#### 2.2.4. Fovea

**Figure 6:**
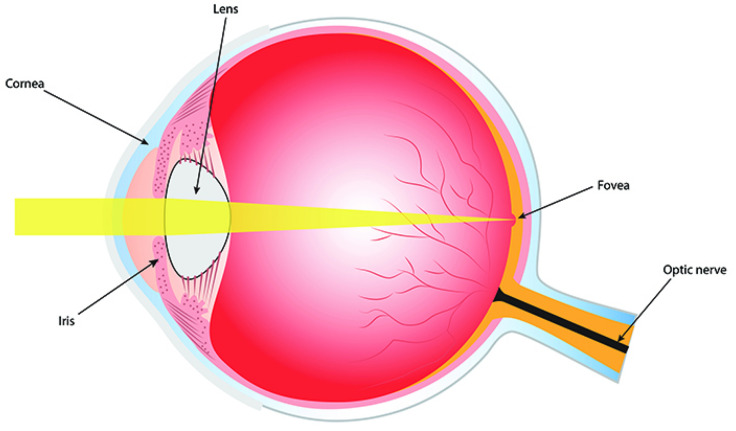
Eye, retina and fovea. Adapted from Wikipedia.

The **fovea** was discovered by Leonardo da Vinci. It is a small pit in the retina which contains mostly cones, see Figure 6. The cental part of the fovea, called the foveola, has a diameter 0.35 mm ∼ 1${}^{{}^{\circ}}$. It consists only from cones packed with maximum density. The fovea occupies 1% of retina, but is projected onto almost 50% of area of the visual cortex. When we fix gaze on a point *A*, the image $\bar{A}$ of this point on retina moved due to the fixation eye movements (FEM), but remains inside fovea.

#### 2.2.5. Inhomogeneity of the Retina and Magnification. Physiological Metric in Retina

The physical metric in retina (considered as a sphere) is standard metric of the sphere. (The distance is described in mm or in degrees). 1 mm = 3.5${}^{{}^{\circ}}$∼ 6 cm at a distance of 1.5 m, 1${}^{{}^{\circ}}$∼ 0.3 mm ∼ 2.5 cm at a distance 135 cm. Apparent diameter of Moon and Sun is 0.5${}^{{}^{\circ}}$ = 0.15 mm = 150 μ. Receptive field of neurons of V1 cortex projected to fovea has diameter 0.25${}^{{}^{\circ}}$–0.7${}^{{}^{\circ}}$ and the area 0.07${}^{{}^{\circ}}$ × 0.15${}^{{}^{\circ}}$∼ 0.12 mm^2^. The receptive field of neurons projected onto the periphery of the retina has a diameter up to 8${}^{{}^{\circ}}$, on average this is 30 times more then in fovea and the RF here contains thousands of rods.

Magnification = distance between two points of V1 cortex which corresponds to 1 mm distance in retina. The cortical magnification in the fovea 1mm ∼ (1/6)${}^{{}^{\circ}}$ = 0.05 mm is 20 times. The cortical magnification in the periphery 1 mm = 6${}^{{}^{\circ}}$ = 1.8 mm is 0.55 times.

Hubel [37] remarked that the structure of retina is very inhomogeneous. He supposed that it is one of the reason, why the information processing in retina is very limited. On the other hand, he emphasized the amazing homogeneity of the cortex V1. It is expressed in the fact that a shift in 2 mm at any point of the cortex corresponds to shift on diameter of the corresponding receptive field in retina. We define the physiological metric in the retina, where the length of a curve is given by the number of receptive fields of neurons along this curve. This metric in the retina is proportional to the physical metric in the cortex. In particular, the diameter of fovea 1${}^{{}^{\circ}}$ corresponds to 6 mm in V1 cortex. (Hubel).

We will discuss a possible application of this metric to choosing of appropriate diffusion kernel for stochastic model of the drift.

#### 2.2.6. Conformal Retinotopic Map from the Retina to the LGN (Lateral Geniculate Nucleus) and to the Visual Cortex V1

After image processing in the retina, the input function is encoded by the firings of ganglion cells. Then it is sent to the LGN and the V1 cortex by the conformal retinotopic mapping, see [38,39]. There are three main pathways from the retina to the V1 cortex: the *P-pathway*, which is responsible for the perception of stable objects, the *M-pathway*, which is important for the perception of moving objects, and the *K-pathway*, important for the color vision. In static models, only the P-pathway is considered, but for dynamic model the *M*-pathway is also very important. M-pathway is more complicated then *P*-pathway, since *M*-neurons are not linear, see [36].

Let (x,y) be the standard coordinates of the tangent plane $T_{F}S^{2}$ of the eye sphere at the center *F* of the fovea. We will consider these coordinates as conformal coordinates on the eye sphere due to the stereographic map with center at the nodal point *N*. It is convenient also to introduce the complex coordinate z=x+iy and the associated polar coordinates r,θ where $z=re^{i\theta }$. In physiology, the coordinate *r* (the geodesic distance to *F*) is called the **eccentricity** and θ the angular coordinate. In appropriate complex coordinate in LGN and the V1 cortex, the retinotopic map is described by a meromorphic function of the form

$$z⟼F\left(z\right)=\log\frac{z+a}{z+b},\;a,b\in R.$$

The module |F(z)| describes the local magnification at a point *z* of the retina (see E. Schwartz [38]).

### 2.3. Functional Architecture of the Primary Visual Cortex: Columns, Pinweels, Simple and Complex Cells, Hypercolumns

The primary visual cortex V1 is a surface of depth 1.5–2 mm which consists of 6 layers. Each layer consists of columns of cells which have approximately the same receptive field. Hubel and Wiesel proposed a classification of V1 cells into simple and complex cells. Simple cells act as Gabor filters (defined by the receptive profile, that is the Gauss function modulated by sin or cos). The most important property of the Gabor filter is that it detects orientation of the contour, crossing its receptive field. There are several versions of the Gabor filters, which measure at the same time other parameters of the stimuli, for example, spatial frequency, phase etc. This means that the Gabor filter is activated only if these parameters take (with some precision) certain values. All simple cells from a **regular column** act as Gabor filters with almost the same center and they detect almost the same orientation of the contour.

A **singular column** called(**pinwheel**) contains simple cells which measure any possible orientation of the contour.

One of the purposes of the eyes movement is to produce the shift of the retinal stimulus such that the contour intersects pinwheels and is detected by their neurons.

#### Hypercolumns of V1 Cortex

Hubel and Wiesel proposed a deep and very productive notion of hypercolumns in V1 cortex. Given a system of local parameters (e.g., orientation, ocular dominance, spatial frequency, temporal frequency, phase etc.). A **lhypercolumn** (or, module) is defined as a minimal collection of (regular) columns, containing simple cells which measure any possible value of these parameters and which is sufficient to detect the local structure of the stimulus. Applying this notion to orientation and ocular dominance, they proposed a famous ice cube model of V1 cortex. Now this notion is applied also for the V2 cortex. Usually, the area of hypercolumns is 1–2 mm^2^.

## 3. Information Processing in Dynamics

### 3.1. The Eye as a Rotating Rigid Ball

From a mechanical point of view, the eye is a rigid ball $B^{3}$ which can rotate around its center *O*. The retina occupies only part of the eye sphere but for simplicity, we identify it with the whole eye sphere $S^{2}=\partial B^{3}$. We will assume that the **eye nodal point** *N* (or **optical center**) belongs to the eye sphere and the opposite point *F* of the sphere at the center of the fovea.

For a fixed position of the head, there is a standard initial position $S_{E}^{2}$ of the eye sphere, described by the canonical orthonormal frame ${\underline{e}}_{0}=\left(i,j,k\right)$, which determines the standard coordinates (X,Y,Z) of the Euclidean space $E^{3}$ with center *O*. We will consider these coordinates as the spatiotopic (or the world-centered) coordinates and at the same time as the head-centered coordinates. Here *i* indicates the standard frontal direction of the gaze, *j* is the lateral direction from left to right which is orthogonal to *i* and *k* is the vertical direction up.

Any other position of the eye is described by an orthogonal transformation $R\in {\mathrm{SO}}_{3}$ which maps the frame ${\underline{e}}_{0}=\left(i,j,k\right)$ into another frame $\left(\underline{e}\right)=\left(e_{1},e_{2},e_{3}\right)=R\left(i,j,k\right)$ where $e_{1}$ is the new direction of the gaze. Recall that any movement R∈SO(3) is a rotation $R_{e}^{\alpha }$ about some axis $e\in S_{E}^{2}$ through some angle α.

#### Definition of a Straight Line by Helmholz

If the frontal plane (orthogonal to the line of sight) is far enough away compared to the size of the eyeball, then the central projection can be considered as a **conformal map**.

H. von Helmholtz gave the following physiological definition of a straight line:

A straight line is a curve $\ell \subset E^{3}$, which is characterized by the following property: when the gaze moves along the curve *ℓ*, the retinal image of *ℓ* does not change.

Indeed, given a straight line ℓ={γ(t)}, let us denote by Π=Π(O,ℓ) the plane through *ℓ* and the center *O* of the eye ball and by *n* its normal vector. Assume that for the standard position $S_{E}^{2}$ of the eye, the gaze is concentrated on the point γ(0), i.e., γ(0)∈Ri. The retina image of *ℓ* belongs to the intersection $\Pi \cap S_{E}^{2}$ between Π and the standard position $S_{E}^{2}$ of the eye sphere. When the gaze moves along γ(t), the eye rotates with the axis *n*. Since at each moment *t* the new position of the eye sphere is $S_{t}^{2}=R_{n}^{t}S_{E}^{2}$, the retina image

$$S_{t}^{2}\cap \Pi =R_{n}^{t}S_{E}^{2}\cap \Pi =R_{n}^{t}\left(S_{E}^{2}\cap \Pi \right)=S_{E}^{2}\cap \Pi$$

remains the same for all *t*.

We will see that saccades correspond to such movements along the straight lines.

### 3.2. Saccades and Fixation Eye Movements: Tremor, Drift and Macrosaccades

#### 3.2.1. Saccades

Eyes participate in different types of movements [40]. We are interested only in saccades and fixation eye movements (FEMs) when the gaze is “fixed” [41].

Saccades are one of the fastest movements produced by the human body. The angular speed of the eye during a saccade reaches up to 700${}^{{}^{\circ}}$/s in humans for great saccades ( 25${}^{{}^{\circ}}$ of visual angle). Saccades to an unexpected stimulus normally take about 200 milliseconds (ms) to initiate, and then last from about 20–200 ms, depending on their amplitude. For amplitudes up to 15${}^{{}^{\circ}}$ or 20${}^{{}^{\circ}}$, the velocity of a saccade linearly depends on the amplitude. Head-fixed saccades can have amplitudes of up to 90${}^{{}^{\circ}}$, but in normal conditions saccades are far smaller, and any shift of gaze larger than about 20${}^{{}^{\circ}}$ is accompanied by a head movement. Most researchers define microsaccades as a small saccades, i.e., saccades with a small amplitude, such that the during a microsaccade the retina image of the point of fixation belongs to the fovea and even foveola, [23]. However in [42], the authors distinguish the small goal-directed voluntary eye movements from microsaccades. They showed that properties of microsaccades are correlated with precursory drift motion, while amplitudes of goal-directed saccades do not dependent on previous drift epochs. Microsaccades represent one of the three types of fixation eye movements.

#### 3.2.2. Fixation Eye Movements (FEM)

The fixation eye movements are responsible for detection of local image structures and consist of tremor, drifts and microsaccades.

**Figure 7:**
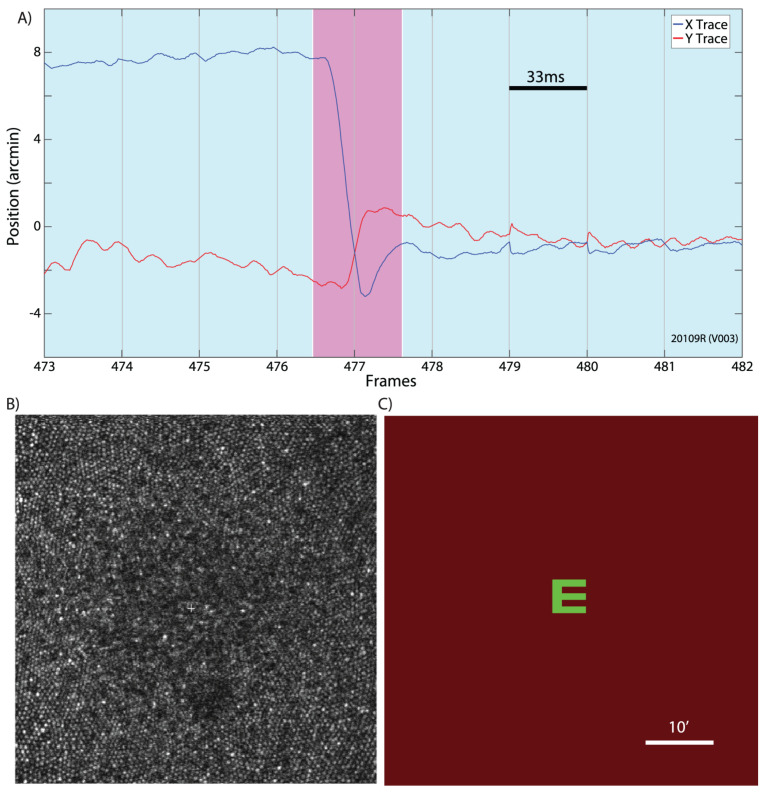
(**A**) An example of an eye trace taken from an AOSLO movie. A microsaccade (magenta background) is clearly distinguishable from the ocular drift (blue background). Gray vertical gridlines demarcate frame boundaries from the AOSLO movie. Each frame is acquired over 33 ms as indicated by the scale bar. (**B**) An example of an image/frame from an AOSLO movie. The cone mosaic can be resolved even at the fovea. (**C**) An example of the AOSLO raster with a green letter E as it would appear to the subject. The small discontinuities in the eye trace at the boundaries between frames 478–479 and 480–481 are likely the result of tracking errors that occur at the edges of the frame. They are infrequent and an example is included here for full disclosure. Errors like this contribute to the peaks in the amplitude spectrum at the frame rate and higher harmonics. All original eye motion traces are available for download. Adapted from [43].

**Tremor** is an aperiodic, wave-like motion of the eyes of high frequency but very small amplitude. We hypothesize that the role of tremor is to increase the width of the contour on the retina, so that it is perceived by several rows of photoreceptors. This will allow also to estimate the value of the gradient along the contour. A detailed study of tremor and its influence on the retina images was made in [43], see Figure 7.

**Drifts** occur simultaneously with tremor and are slow motions of eyes, in which the image of the fixation point for each eye remains within the fovea. Drift is an involuntary stochastic process. However, the stochastic characteristics of the drift may depend on the local structure of the stimulus. Drifts occur between the fast, jerk-like, linear **microsaccades**. The main property of the FEMs is that during FEM the retina image of the point of fixation remains in the fovea and even the foveola [23]. The following Table 1 indicates the main characteristics of the FEM.

**Figure 8:**
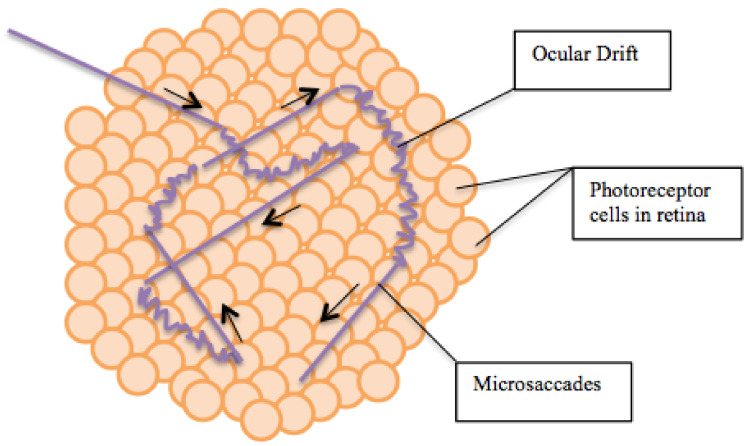
Microsaccades and Ocular Drifts. Adapted from Wikipedia https://commons.wikimedia.org/wiki, CC-BY.

Per 1 s tremor moves on 1–1.5 diameters of the fovea cone, drift moves on 10–15 diameters, microsaccads moves on 15–300 diameters, see Figure 8.

#### 3.2.3. The Role of Fixation Eye Movements

The papers by M. Rucci and his collaborators [21,22,23,24,25] contain very useful information about different characteristics of fixation eye movements and a detailed analysis of the role of FEM in vision. In a survey [23], the authors critically revised three main hypotheses about the role of microsaccades (MS) in vision:

(1) the maintenance of accurate fixation;
(2) the prevention of image fading due to fast adaptation of retinal photoreceptors;
(3) vision of fine spatial detail.

They gave many very convincing arguments in support of the hypotheses (1) and (3) and 10 arguments against the hypothesis (2). We add here only one additional argument against (2). Support that before the MS a retinal photoreceptor in fovea received light signal from stimulus *A*. After the MS, it will receive a signal from another stimulus *B*, which can be even brighter. Why this will prevent the photoreceptor from adaptation?

We mention also one geometric argument why FEM are useful for vision. In monocular vision, provided that the position of eye is fixed, the retina gets information only from the 2-dimensional Lagrangian submanifold $L\left(N\right)=\left\{\ell ∋N\right\}≃RP^{2}$ of the 4-dimensional space of lines $L(E^{3})$ consisting of lines incident to the eye nodal point *N*. The space of lines is naturally identified with the (co)tangent bundle $T^{*}S^{2}≃TS^{2}$ of the unit sphere. It is a symmetric pseudo-Kähler manifold of neutral signature (2,2). When the eye moves with a small amplitude, the retina gets information from a neighborhood of this 2-surface L(F) in the 4-manifold $L(E^{3})$.

M. Poletti and M. Rucci [23] gave evidence that during natural vision the microsaccades can not be regarded as a random process. Their characteristics depend on the scene. Moreover, the ability to control microsaccades plays an important role in performing different fine work, like reading, threading a needle, playing some sports (e.g., table tennis), etc. However, it seems plausible that in some cases MS can be considered as random processes. For example, when contemplating the sea, the blue sky and similar homogeneous scenes, it can be assumed that microsaccades make a random walk. Perhaps the pleasure that a person feels when contemplating such scenes is due to the fact that the eyes get rid of the difficult work of finding new targets for microsaccades.

#### 3.2.4. Remapping and Shift of the Receptive Fields (RFs)

In a seminal paper, J.-R. Duhamel, C.L. Colby and M.E. Goldberg [45] described the **shift of receptive field** of many neurons in macaque lateral intraparietal area (LIP), which shows that the visual neurons of these systems get information about the retina images of their future receptive fields. This is one of the most remarkable discoveries of neurophysiology of vision at the end of the 20th century.

Assume that the RF of a neuron before a saccade covers the retina image $\bar{A}$ of a point *A* and after the saccade the retina image $\bar{B}$ of another point *B*. Then 100 ms before the saccade, the neuron detects stimuli at the locations $\bar{B}$. This process constitutes a **remapping** of the stimulus from the retina coordinates with the initial fixation point *A* to those of the future fixation point *B*. The process is governed by a copy of the motor command (corollary discharge).

For a long time, it had been assumed that the presaccadic shift of the receptive field (RF) from $\bar{A}$ to $\bar{B}$ is an anticipation of the retinal consequences of the saccade, which randomly changes the gaze direction and the RF of the neurons to $\bar{B}$. Since *any* point $\bar{B}$ of the retina can be a new position of the receptive field, this means that the information about the visual stimulus at the point $\bar{B}$ can be transmitted to neurons with receptive field at the point $\bar{A}$. This seems very doubtful, since the number of neurons pairs is too big. The solution was proposed by M. Zirnsac and T. Moore [46]. They conjectured that the presaccadic shift of RF is a part of a process of remapping and reflects the selection of the targets for the saccades. Some local area of a higher center of the visual system has information about visual stimulus concentrated at $\bar{A}$ and about other points of the retina. It uses this information to choose a new saccadic target $\bar{B}$. Just before the saccade, it sends the information about the visual stimulus at the retinal point $\bar{B}$ to neurons with presaccadic receptive field at $\bar{A}$. After saccades, the real RF of these neurons cover the retina stimulus $\bar{B}$. Then the visual system use information from these neurons to corrects the presaccadic information. In the last section, we propose a mechanism of realization of such presaccadic remapping.

#### 3.2.5. Oculomotor System, Corollary Discharge and Stability Problem

In dynamic, the retinal photoreceptors are not the only source of visual information. The important part of information about eyes movements is coded in oculomotor system. A copy of motor commands, which control eyes movements, the **corollary discharge (CD) or efference copy**, is sent from the sensorimotor region through the MD thalamus to the frontal cortex. The mechanism of interaction of CD information with information from retinal receptors processed in the visual cortex is not well known. It is very important for solution of the stability problem, i.e., explanation of the compensation mechanism for shift of stimuli on the retina caused by eye movements, such that a stable stimuli will be perceived as stable, see [26,27,28]. Clearly, it must be very strong synchronization between corollary discharge and the presentation of the retina input function in visual cortex.

The stability problem was first formulated in the eleventh century by the Persian scholar Abu’Ali al-Hasan ibn al-Hasan ibn al-Haytham (latinized, Alhazen) and was discussed by Descartes, Helmholtz, Mach, Sherrington and many others scientists.

### 3.3. The Geometry of the Quaternions

Now we recall the basic facts about quaternions and the Hopf bundle, which are we need for reformulation of Donders’ and Listing’s laws in terms of Listing’s section of the Hopf bundle.

Let H=R4=R1+ImH=R1+E3 be the algebra of quaternions with the unit 1, where the space $E^{3}$ of the imaginary quaternions is the standard Euclidean vector space with the orthonormal basis (i,j,k) and the product ab of two elements from *E* is the sum of their scalar product and the cross-product:

ab=〈a,b〉+a×b.

The group

$$H_{1}=\{q=q_{0}1+q^{\prime }{,\;|q|}^{2}:=q_{0}^{2}+|q^{\prime }{|}^{2}=1\}=S^{3}$$

of unit quaternions are naturally identified with the three dimensional sphere $S^{3}$ and its Lie algebra is the algebra $E^{3}=R^{3}$ of imaginary quaternions with the cross-product as the Lie bracket.

Denote by

$$L:H_{1}\to SO\left(R^{4}\right),\;a\mapsto L_{a},\;L_{a}q=aq,\;q\in H$$

the (exact) left representation and by

$$R^{-1}:H_{1}\to SO\left(R^{4}\right),\;a\mapsto R_{a}^{-1},\;R_{a}^{-1}q=q\bar{a},\;q\in H$$

the (exact) right representation, which commutes with the left representation. They define the representation

$$L\times R^{-1}:H^{1}\times H^{1}\to SO\left(4\right)=\left(L_{H_{1}}\times R_{H_{1}}\right)/Z_{2}$$

with the kernel $Z_{2}=\left\{\pm 1\right\}$.

The representation

$$\mathrm{Ad}:H_{1}\to SO\left(4\right),\;a\mapsto {\mathrm{Ad}}_{a}.\;{\mathrm{Ad}}_{a}q=aq\bar{a}$$

is called the **adjoint representation**. It has the kernel $Z_{2}=\left\{\pm 1\right\}$, acts trivially on the real line R1 and defines the isomorphism $H_{1}/Z_{2}={\mathrm{Ad}}_{H_{1}}=SO\left(E^{3}\right)=SO\left(3\right)$ which shows that the group $H_{1}=S^{3}$ is the universal covering of the orthogonal group SO(3). The standard scalar product $\langle q,q\rangle =q\bar{q}$ in H, where for $q=q_{0}1+q^{\prime }$ the $\bar{q}:=q_{0}-q^{\prime }$ is the conjugated quaternion, induces the standard Riemannian metric of the unit 3-sphere $S^{3}=H_{1}$, which is invariant with respect to the (transitive) actions of the group $L_{H_{1}}\times R_{H_{1}}^{-1}$. The group ${\mathrm{Ad}}_{H}$ preserves the points 1,−1 (which will be considered as poles of $S^{3}$) and acts transitively on the equator $S_{E}^{2}:=S^{3}\cap E^{3}$, which is the standard Euclidean unite sphere of the Euclidean space $E^{3}$. The geodesics of $S^{3}$ are the great circles (the intersections of $S^{3}$ with 2-subspaces of $H=R^{4}$).

The following simple facts are important for us and we state them as

**Lemma** **1.**

 *(i)*  *Any point $a\in S^{3}$ different from ±1 belongs to unique 1-parameter subgroup $g_{a}=\mathrm{span}\;\left(1,a\right)\cap S^{3}$ (the meridian) and can be canonically represented as*
$$a=e^{\psi v}:=\cos\psi +\sin\psi \;v,\;0\leq \psi <\pi /2,vs.\in S_{E}^{2},$$
  *where $v=pr_{S_{E}^{2}}a$ is the closest to a point of the equator.*
 *(ii)*  *Points $v\in S_{E}^{2}\setminus \pm 1$ bijectively corresponds to oriented 1-parameter subgroups*
$$g_{v}\left(t\right):=e^{tv}=\cos t+\sin t\;v$$
  *of $H_{1}$, parametrized by the arclength.*
 *(iii)*  *Any orbit $\gamma \left(t\right)=g_{v}\left(t\right)a,\;a\in S^{3}$ of the left action of an one-parameter subgroup γ(t) (as well as the right action) is a geodesic of the sphere $S^{3}$. All geodesics are exhausted by such orbits.*

#### 3.3.1. The Adjoint Action of the Group $H_{1}$

**Lemma** **2.**

 *(i)*  *The 1-parameter subgroup $g_{v}\left(t\right)=e^{tv}$ of $H_{1}$ generated by a unit vector $v\in S_{E}^{2}\subset H_{1}$ acts on the sphere $S_{E}^{2}$ as the 1-parameter group $R_{v}^{2t}$ of rotation w.r.t. the axe v:*
$$Ad_{g_{v}\left(t\right)}=R_{v}^{2t}.$$
 *(ii)*  *More generally, let*
$$\gamma \left(t\right)=g_{v}\left(t\right)a\subset S^{3}=H_{1}$$
  *be a geodesic of $S^{3}$, considered as the orbit of an 1-parameter subgroup $g_{v}\left(t\right)$. Then for $x\in S_{E}^{2}$ the adjoint action of the curve γ(t) is given by*
$${\mathrm{Ad}}_{\gamma (t)}x={\mathrm{Ad}}_{\gamma (t)}x={\mathrm{Ad}}_{g_{v}\left(t\right)}x^{a}=R_{v}^{2t}\left(x^{a}\right),\;\mathrm{where}\;\;x^{a}:={\mathrm{Ad}}_{a}x=ax\bar{a}.$$
*In other words, the orbit ${\mathrm{Ad}}_{\gamma (t)}x$ is the circle, obtained from the point $x^{a}$ by action of the group ${\mathrm{Ad}}_{g_{v}}\left(t\right)=R_{v}^{2t}$ of rotations w.r.t. the axe v.*

**Proof.** 

(i) The adjoint image $Ad_{g_{v}\left(t\right)}$ of the one-parameter subgroup is an one-parameter subgroup of $SO(E^{3})$, which preserves the vector $v\in S_{E}^{2}$, hence the group $R_{v}$ of rotation w.r.t. *v*. To calculate the angle of the rotation, we apply ${\mathrm{Ad}}_{g_{v}\left(t\right)}$ to a vector $u\in S_{E}^{2}$, which anticommutes with *v*, as follows
  $${\mathrm{Ad}}_{g_{v}\left(t\right)}u=e^{tv}ue^{-tv}=e^{2tv}u=\cos2tu+\sin2t\;vu.$$
  This shows that ${\mathrm{Ad}}_{g_{v}\left(t\right)}=R_{v}^{2t}$.
(ii) follows from (i) and the following calculation
  $${\mathrm{Ad}}_{\gamma (t)}x=g_{v}\left(t\right)ax\bar{a}{\bar{g}}_{v}\left(t\right)={\mathrm{Ad}}_{g_{v}\left(t\right)}ax\bar{a}={\mathrm{Ad}}_{g_{v}\left(t\right)}x^{a}=R_{v}^{2t}x^{a}.$$

□

#### 3.3.2. The Hopf Bundle and Listing’s Sphere

The Hopf bundle is defined as the natural projection

$$\chi :S^{3}=H_{1}\to S_{E}^{2}=S^{3}/SO_{2},\;\;\;q\mapsto {\mathrm{Ad}}_{q}i=qi\bar{q}$$

of $H_{1}=S^{3}$ to the ${\mathrm{Ad}}_{H_{1}}$-orbit $S_{E}^{2}=Ad_{H_{1}}i$ of the point *i*.

The base sphere $S_{E}^{2}=S^{3}\cap E^{3}$ is called the Euclidean 2-sphere. The points i,−i will be considered the north and south poles of $S_{E}^{2}$. We denote by $S_{E}^{1}=\left\{p=\cos\theta \;j+\sin\theta \;k\right\}$ the equator of $S_{E}^{2}$.

The Hopf bundle is a non trivial bundle and has no global section. However, by removing just one point −i with the preimage $S_{E}^{1}$ from the base sphere $S_{E}^{2}$, we will construct the canonical section

$$s:{\tilde{S}}_{E}^{2}=S_{E}^{2}\setminus \left\{-i\right\}\to {\tilde{S}}^{3}=S^{3}\setminus S_{E}^{1}.$$

of the bundle

$$\chi :{\tilde{S}}^{3}\to {\tilde{S}}_{E}^{2}$$

over the punctured sphere ${\tilde{S}}_{E}^{2}$.

**Figure 9:**
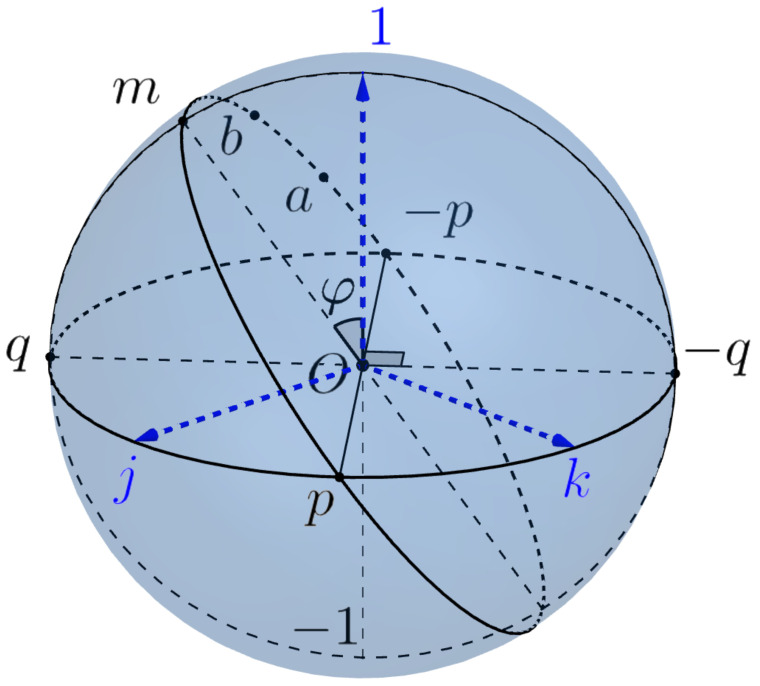
Listing’s sphere.

First of all, we define Listing’s sphere and Listing’s hemisphere, which play a central role in the geometry of saccades. The **Listing’s sphere** is intersection $S_{L}^{2}=S^{3}\cap i^{⊥}$ of the 3-sphere with the subspace $i^{⊥}=\mathrm{span}\;\left(1,j,k\right)$ spanned by vectors 1,j,k. In other words, it is the equator of the 3-sphere $S^{3}$ w.r.t. the poles ±i, see Figure 9.

We consider the point 1 (resp., **−1**) as north (resp. south) pole of Listing’s sphere and denote by $S_{L}^{+}$ (resp., $S_{L}^{-}$) the open north (resp., south) hemisphere and by ${\bar{S}}_{L}^{+}$ (resp., ${\bar{S}}_{L}^{-}$) the closed hemisphere. Note that the equator $S_{L}^{1}$ of Listing’s sphere coincides with the equator $S_{E}^{1}$ of the Euclidean sphere $S_{E}^{2}$.

#### 3.3.3. Geometry of Listing’s Hemisphere $S_{L}^{+}$

We consider Listing’s sphere as the Riemannian sphere with the induced metric of curvature 1 equipped with the polar coordinates (r,θ) centered at the north pole 1. The geodesics of $S_{L}^{2}$ are big circles. Any point $a=e^{rp}=\cos r1+\sin r\;p\neq \pm 1$ of $S_{L}^{2}$ belongs to the unique 1-parameter subgroup $g_{a}\left(t\right)=e^{ta}=\cos t1\;+\sin ta$ of $H_{1}$.

Any point $a\in S_{L}^{+}$, different from 1, can be canonically represented as

$$a=e^{rp}:=\cos r1+\sin r\;p,\;\;p=\cos\theta \;j+\sin\theta \;k\in S_{L}^{1}$$

where 0<r<π/2 is the polar radius (the geodesic distance to the pole 1 (such that φ:=π/2−r is the geographic latitude) and 0≤θ<π is the geographic longitude of the point *a*. The point $p=pr_{S_{L}^{1}}a$ is the geodesic projection of *a* to the equator, i.e., the closest to *a* point of the intersection of $g_{a}\left(t\right)=\gamma_{a,1}$ with the equator $S_{L}^{1}$.

Note that the coordinate lines θ=const are big circles (meridians), in particular, θ=0 is zero ("greenwich") meridian and the coordinate lines φ=const are parallels. The only geodesic parallel is the zero parallel, i.e., the equator $S_{L}^{1}$.

The open Listing’s hemisphere $S_{L}^{+}$ is geodesic convex. This means that any two distinct points $a,b\in S_{L}^{+}$ determine a unique (oriented) geodesic $\gamma_{a,b}$ of the sphere $S_{L}^{2}$ and are joined by a unique geodesic segment $ab\subset S_{L}^{+}$.

##### Canonical Parametrization of Geodesics $\gamma_{a,b}\subset S_{L}^{2}$

Let $a,b\in S_{L}^{+}$ be two distinct points and $\gamma_{a,b}$ the oriented geodesic. Denote by *p* the first point of intersection of γ(a,b) with the equator $S_{L}^{1}$.

If $1\in \gamma_{a,b}$ then the geodesic is an 1-parameter subgroup and

$$\gamma_{a,b}=e^{tp}=\cos t1+\sin\;p$$

is its canonical parametrization.

If $1\notin \gamma_{a,b}$, the unique top point $m\in \gamma_{a,b}$, with the maximal latitude φ has the form $m=e^{\varphi q}$ where $q=pr_{S_{L}^{1}}m\in S_{L}^{1}$ is the geodesic projection of *m* to $S_{L}^{1}$ and 〈p,q〉=0, hence q=±pi.

Then

$$\gamma_{a,b}=\gamma_{p,m}=\left\{\cos t\;p+\sin t\;m=e^{tv}p\right\},\;v=m\bar{p}=-mp,$$

where $vs.=m\bar{p}=-\cos\varphi \;p+\sin\varphi \;pq\in S_{E}^{2}$ and pq=±i, is the **canonical parametrization** of the geodesic $\gamma_{a,b}$.

The intersection $\gamma_{a,b}^{+}=\gamma_{a,b}\cap S_{L}^{+}$ of the geodesic with the Listing hemisphere $L_{L}^{+}$ is called the **Listing’s semicircle**.

#### 3.3.4. Properties of the Restriction of the Hopf Map to Listing’s Sphere

**Theorem** **1.** *The restriction $\chi :S_{L}^{2}\to S_{E}^{2}$ of the Hopf map χ to the Listing sphere is a branch $Z_{2}$ covering. More precisely*

 *(i)*  *It maps the poles ±1 of the sphere $S_{L}^{2}$ into the pole i of the sphere $S_{E}^{2}$ and the equator $S_{L}^{1}$ into the south pole $-i=\chi (S_{L}^{1})$.*
 *(ii)*  *Any different from 1 point $a\in S_{L}^{2}$ belongs to a unique 1-parameter subgroup $g_{a}=e^{t^{\prime }a}$ (the meridian of Listing’s sphere) which can be written as $g_{a}=g_{p}=e^{tp}$ where $p=pr_{S_{L}^{1}}a=\cos\theta \;j+\sin\theta \;k\in S_{L}^{1}$ is the equatorial point of $g_{a}$.*
*The map $\chi :g_{a}\to S_{p}^{1}$ is a locally isometric $Z_{2}$ covering of the meridian $g_{a}=\gamma_{p,1}$ of Listing’s sphere $S_{L}^{2}$ onto the meridian $S_{p}^{1}$ of the Euclidean sphere $S_{E}^{2}$ through the point $p\in S_{E}^{1}$. The restriction of χ to the semicircle $g_{a}\cap S_{L}^{+}$ is a diffeomorphism.*
 *(iii)*  *More generally, let $\gamma_{a,b}=\gamma_{p,m}=\left\{e^{tv}p\right\},\;v=m\bar{p}$ be a geodesic through points $a,b\in S_{L}^{+}$ with the canonical parametrization*
$$\gamma_{p,m}\left(t\right)=\cos t\;p+\sin t\;m,\;m=e^{\varphi q}=\cos\varphi \;1+\sin\varphi \;q.$$
*It is the orbit $e^{tv}p$ of 1-parameter group $e^{tv}$,*
$$vs.=m\bar{p}=-\cos\varphi \;p+\sin\varphi \;pq\in S_{E}^{2}$$
  *and the Hopf mapping χ maps it into the orbit*
$$S_{v}^{1}:=\left\{{\mathrm{Ad}}_{e^{tv}}\left(-i\right)=\right\}=\left\{R_{v}^{2t}\left(-i\right)\right\}\subset S_{E}^{2},$$
  *of the 1-parameter group of rotations $R_{v}^{2t}$. In other words, the circle $S_{v}^{1}:=\chi \left(\gamma_{p,m}\left(t\right)\right)$ is obtained by rotating the point −i about the axis $v\in S_{E}^{2}$.*
 *(iv)*  *The restriction of the map χ to the Listing hemisphere $S_{L}^{+}$ is a diffeomorphism $\chi :S_{L}^{+}\to {\tilde{S}}_{E}^{2}$.*

**Proof.**  (i)–(ii) follow from the remark that quaternions ±1 commute with *i* and the quaternions from $S_{L}^{1}$ anticommute with *i*. Hence ${\mathrm{Ad}}_{e^{tp}}i=e^{2tp}i=ie^{-2tp}$ for $p\in S_{L}^{1}$.

(iii) We calculate

$$\chi \left(\gamma_{p,m}\left(t\right)\right)=\chi \left(e^{tv}p\right)=e^{tv}pi\bar{p}e^{-tv}=-e^{tv}ie^{-tv}=-e^{2tv}i=R_{v}^{2t}\left(-i\right).$$

(iv) follows from (ii) or from Lemma 2. □

#### 3.3.5. Listing Section

According to the Theorem, the Hopf map defines a diffeomorphism

$$\chi :S_{L}^{+}\to {\tilde{S}}_{E}^{2}:=S_{E}^{2}\setminus \left\{-i\right\}$$

$$a=e^{tp}\mapsto A:=\chi \left(a\right)=e^{2tp}i=R_{p}^{2t}i=\cos2t\;i+\sin2t\;q,\;\;q:=pi=-ip.$$

Since the preimage $\xi^{-1}\left(-i\right)=S_{L}^{1}$ is the equator of Listing’s sphere $S_{L}^{2}$, the inverse map

$$s:=\chi^{-1}:{\tilde{S}}_{E}^{2}\to S_{L}^{+}\subset S^{3}$$

$$A:=e^{2tq}i=\cos2t\;i+\sin2t\;q=\to a:=s\left(A\right)=e^{tp}=\cos t+\sin t\;p,$$

where $q\in S_{E}^{1}=S_{L}^{1},\;q=pi$ is a section of the principal bundle

$$\chi :{\tilde{S}}^{3}:=S^{3}\setminus S_{L}^{1}\to {\tilde{S}}_{E}=S_{E}^{2}\setminus \left\{-i\right\}.$$

We call the section *s* the **Listing section**.

### 3.4. The Physiological Interpretation: Donders’ and Listing’s Laws and Geometry of Saccades

We use the developed formalism to give an interpretation of Donders’ and Listing’s laws and to study the saccades and drifts.

**Figure 10:**
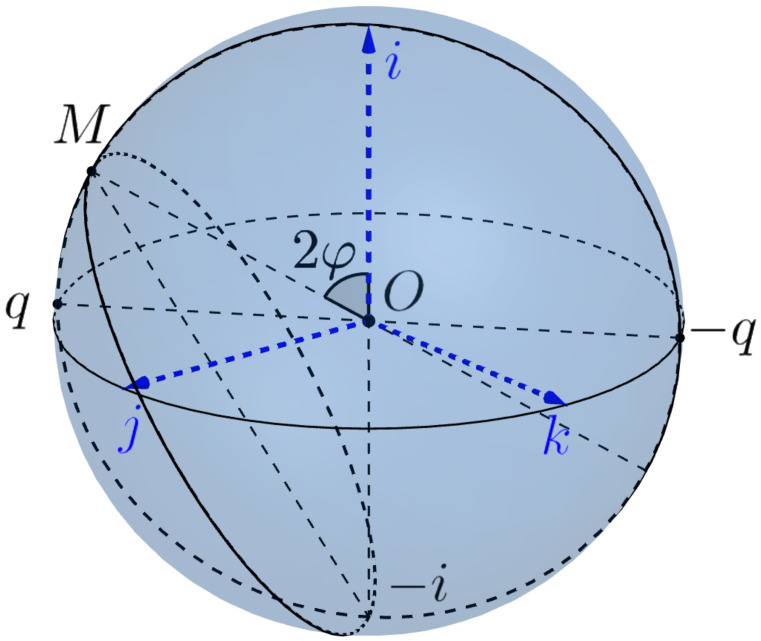
The eye sphere.

We consider the Euclidean sphere SE2⊂ImH=R3 as the model of the eye sphere, see Figure 10, (the boundary of the eye ball B3⊂R3=ImH) with the center at the origin 0. We assume that the head is fixed and the standard basis ${\underline{e}}_{0}=\left(i,j,k\right)$ determines the standard initial position of the eye, where the first vector *i* (the **gaze vector**) indicates the standard frontal direction of the gaze, the second vector *j* gives the lateral direction from right to left and *k* is the vertical direction up.

The coordinates (X,Y,Z) associated with the standard basis are the head-centered and spatiotopic (or world-centered) coordinates. A general position of the eye, which can rotate around the center 0 is determined by the orthonormal moving (retinatopic) frame $\underline{e}=\left(e_{1},e_{2},e_{3}\right)$, which determine the (moving) retina-centered coordinates (x,y,z).

The configuration space of the rotating sphere is identified with the orthogonal group SO(3), an orthogonal transformation *R* define the frame

$$\underline{e}=\left(e_{1},e_{2},e_{3}\right)=R{\underline{e}}_{0}=R\left(i,j,k\right).$$

It is more convenient to identify the configuration space with the group $H_{1}=S^{3}$ of unit quaternions, which is the universal cover of SO(3). The corresponding $Z_{2}$-covering is given by the adjoint representation

$$\mathrm{Ad}:H_{1}\to SO\left(3\right)=H_{1}/\left\{\pm 1\;\right\},\;a\mapsto {\mathrm{Ad}}_{a}.$$

A unit quaternion $a\in H_{1}$ gives rise the orthogonal transformation ${\mathrm{Ad}}_{a}\in SO\left(E^{3}\right)$ and the frame $\underline{e}={\mathrm{Ad}}_{a}\underline{e_{0}}={\mathrm{Ad}}_{a}\left(i,j,k\right)$ which defines the new position of the eye. We have to remember that opposite quaternions $a,-a\in H_{1}$ represent the same frame and the same eye position. Note that a direction of the gaze $e_{1}$ determines the position $\underline{e}=\left(e_{1},e_{2},e_{3}\right)$ of the eye up to a rotation w.r.t. the axe $e_{1}$. Such rotation is called the **twist**.

**Donders’ law** states that if the head is fixed, then there is no twist. More precisely, the position of the gaze $A=e_{1}\in S_{E}^{2}$ determines the position of the eye, i.e., there is a (local) section $s:S_{E}^{2}\to S^{3}$ of the Hopf bundle

$$\chi :S^{3}=H_{1}\to S_{E}^{2}={\mathrm{Ad}}_{H_{1}}i.$$

In other words, the admissible configuration space of the eye is two-dimensional. Physiologists were very puzzled by this surprise. Even the great physiologist and physicist Hermann von Helmholtz doubted the justice of this law and recognized it only after their own experiments. However, from the point of view of the modern control theory, it is very natural and sensible. The complexity of motion control in 3-dimensional configuration space compared to control on the surface is similar to the difference between piloting a plane and driving a car.

Listing’s law specifies the section *s*. In our language, it can be stated as follows.

**Listing’s law.** The section of Donder’s law is the Listing’s section

$$s=\chi^{-1}:{\tilde{S}}_{E}^{2}=S_{L}^{2}\setminus \left\{-i\right\}\to S_{L}^{+}\subset S^{3}$$

$$A=e_{1}=\cos ti+\sin tq=e^{tq}i\mapsto a=s\left(A\right)=e^{(t/2)p}$$

where $p=q\bar{i}=iq$, which is the inverse diffeomorphism to the restriction

$$\chi :S_{L}^{+}\to {\tilde{S}}_{E}^{2}$$

$$a=e^{tp}\mapsto a:=\chi \left(a\right)={\mathrm{Ad}}_{e^{tp}}i=e^{2tp}i=R_{p}^{2t}i=\cos2ti+\sin2tq,\;q=pi.$$

of the Hopf projection to Listing’s hemisphere.

In other words, a gaze direction $A=e_{1}=\cos ti+\sin tq\in {\tilde{S}}_{E}^{2}$ determines the position $\underline{e}=\left(e_{1},\;e_{2}\;e_{3}\right)$ of the eye as follows

$$\underline{e}={\mathrm{Ad}}_{s_{e_{1}}}{\underline{e}}_{0}={\mathrm{Ad}}_{e^{(t/2)p}}\left(i,j,k\right),\;p=q\bar{i}\in S_{L}^{1}=S_{E}^{1}.$$

#### Saccades

We define a **saccade** as a geodesic segment $ab\subset S_{L}^{+}$ of the geodesic semicircle $\gamma_{a,b}^{+}=\gamma_{a,b}\cap S_{L}^{2}$. Recall that the semicircle $\gamma_{a,b}^{+}=\gamma_{p,m}^{+}$, (where *p* is the first point of the intersection of the oriented geodesic $\gamma_{a,b}$ with the equator $S_{L}^{1}$, $m=e^{\varphi q}$ is the top point of $\gamma_{a,b}^{+}$ and *q* is the equatorial point of the meridian of the point *m*), has the natural parametrization

$$\gamma_{p,m}^{+}\left(t\right)=\left\{\cos t\;p+\sin t\;m=\left(\cos t+\sin t\;m\bar{p}\right)p=e^{tv}\left(p\right)\right\},\;0<t<\pi$$

where $v=m\bar{p}=\left(\cos\varphi \;p+\sin\varphi \;q\right)\bar{p}=-\cos\varphi +\sin\varphi \;\left(q\bar{p}\right)$. We may chose the vector *q*, defined up to a sign, such that $q\bar{p}=i$.

The image

$$\chi \left(\gamma_{a,b}^{+}\right)={\mathrm{Ad}}_{e^{tv}}pi={\mathrm{Ad}}_{e^{tv}}pi\bar{p}={\mathrm{Ad}}_{e^{tv}}\left(-i\right)=R_{v}^{2t}\left(-i\right)=S_{v}^{1}\subset \tilde{S_{E}^{2}}$$

is the circle $S_{v}^{1}$ (without the point −i), obtained by the rotating of the point −i with respect to the axe Rv, or, in other words, it is the section of the punctured sphere ${\tilde{S}}_{E}^{2}$ by the plane −i+span(A+i,B+i) with the normal vector v∈R(A+i)×(B+i), where A=χ(a),B=χ(b). The segment $AB\subset S_{v}^{1}$ is the **gaze curve**, the curve, which describes the evolution of the gaze during the saccade $ab\subset \gamma_{a,b}^{+}$.

The natural question arises. If the gaze circle $S_{v}^{1}$ is not a meridian, it is not a geodesic of ${\tilde{S}}_{E}^{2}$ and the gaze curve $AB\subset S_{v}^{1}$ is not the shortest curve of the sphere, joint *A* and *B*. Why the eye does not rotate such that the gaze curve AB is not the geodesic?

The answer is the following. If all gaze curves during saccades would be geodesics, then we get the twist and the configuration space of the eye becomes three-dimensional. Assume that the gaze curve of three consecutive saccades is a geodesic triangle ABC which starts and finishes in the north pole A=i. Since the sphere is a symmetric space, moreover, the space of constant curvature, the movements along a geodesic induce a parallel translation of tangent vectors. This implies that after saccadic movements along the triangle, the initial position $e_{0}=\left(i,j,k\right)$ of the eye will rotates w.r.t. the normal axe *i* on the angle α which is proportional to the area of the triangle. Hence, a twist will appear.

Fortunately, since the retina image of the fixation point during FEM remain in the fovea with the center at −i, the gaze curve remains in a small neighborhood of the standard position *i*. In this case, the deviation of the gaze curve AB during MS from the geodesic will be very small. This is important for energy minimization, since during wakefulness, 2–3 saccades occur every second. Hence more than 100,000 saccades occur during the day.

Consider the stereographic projection $\pi_{-i}:{\tilde{S}}_{E}^{2}\to T_{i}S_{E}^{2}$ of the sphere ${\tilde{S}}_{E}^{2}$ onto the tangent plane at the point $T_{i}S^{2}$. It is a conformal diffeomorphism, which maps any gaze circle $S_{v}^{1}\subset {\tilde{S}}_{E}^{2}$ onto a straight line and any gaze curve AB of a saccade ab onto an interval $A^{\prime }B^{\prime }=\pi \left(AB\right)=\pi \left(A\right)\pi \left(B\right)$ where $A^{\prime }$ is the point of the intersection of the tangent plane $T_{i}S_{E}^{2}=i+\mathrm{span}\;\left(j,k\right)$ with the line −i+R(A+i) and similar for $B^{\prime }$. More precisely, $A^{\prime }=-i+\frac{2}{1+\cos\psi }\left(A+i\right)$ where A=cosψi+sinψq. The spherical n-gon $A_{1}A_{2}\cdots A_{n}$, formed by gaze curves $A_{1}A_{2},\cdots ,A_{n}A_{1}$ of saccades, maps into the n-gone $A_{1}^{\prime }\cdots A_{n}^{\prime }$ on the plane, such that the angles between adjacent sides are preserved.

### 3.5. Listing’s Section and Fixation Eye Movements

Below we propose an approach to description of information processing in dynamics.

#### 3.5.1. Retinotopic Image of a Stable Stimulus during Eye Movements

Recall that the direction $N=e_{1}$ of the gaze determines the position $a=s\left(N\right)\in S_{L}^{+}$ of the eye, which determines the frame $\underline{e}=\left(e_{1},e_{2},e_{3}\right):={\mathrm{Ad}}_{a}{\underline{e}}_{0}={\mathrm{Ad}}_{a}\left(i,j,k\right)$ and associated retinotopic coordinates.

Let the eye look for some time [0,T]] at a stationary surface, for example, at a plane Π, and the gaze describes a curve $N\left(t\right)\subset S_{E}^{2}$ and hence is directed to the points A(t):=RN(t)∩Π of the stimulus Π. Then the eye position is defined by the curve a(t)=s(N(t)). We call a(t) **Listing’s curve**.

The retinal image of the points A(t) forms the curve $\bar{A}\left(t\right):=-N\left(t\right)$.

Moreover, if $\bar{B}\left(0\right)$ it the retinal image of a point B∈Π at t=0, then due to eye movement, the retinal image $\bar{B}\left(t\right)$ of the same point *B* at the moment *t* will be

$$\bar{B}\left(t\right)={\mathrm{Ad}}_{\bar{a}\left(t\right)}\bar{B}\left(0\right),\;\bar{a}=a^{-1}.$$

Hence the retinal curve $\bar{B}\left(t\right)$ is the retinal image of the external point *B*. Indeed, in retinotopic coordinates, the eye is stable and the external plane Π is rotating in the opposite direction and at the moment *t* take the position $\Pi \left(t\right):={\mathrm{Ad}}_{\bar{a}}\Pi$. The point $\bar{B}\left(t\right)\in \Pi t$ is the new position of the point ${\mathrm{Ad}}_{a(t)}B\left(t\right)=B\left(0\right)$.

#### 3.5.2. *n*-Cycles of Fixation Eye Movements

We define a **fixation eye movement *n*-cycle** as a FEM which starts and finishes at the standard eye position $a_{0}=1$ and consists of *n* drifts $\delta_{k}=\delta \left(a_{k-1},a_{k-1}^{\prime }\right),\;k=1,\cdots ,n$ and *n* microsaccades $S_{k}=a_{k-1}^{\prime }a_{k}$ between them. We will assume that MSs are instantaneous movements and occur at times $T_{1},T_{2},\cdots ,T_{k}=T$. Then the corresponding Listing’s curve can be written as

$$\delta \left(a_{0},a_{0}^{\prime }\right),a_{0}^{\prime }a_{1},\delta \left(a_{1},a_{1}^{\prime }\right),a_{1}^{\prime }a_{2},\cdots ,\delta \left(a_{n-1},a_{n-1}^{\prime }\right),a_{n-1}^{\prime }a_{n},\;a_{0}=a_{n}=1.$$

We associate with *n*-cycle the spherical polygon $P\subset S_{L}^{+}$ with 2n vertices (2n-gone)

$$P=\left(a_{0},a_{0}^{\prime },a_{1},a_{1}^{\prime },\cdots ,a_{k-1},a_{k-1}^{\prime },a_{k}\right),\;\;a_{0}=a_{k}=1.$$

The sides $\left(a_{k-1}^{\prime },a_{k}\right)$ represent saccades $S_{k}=a_{k-1}^{\prime }a_{k}$ and the sides $\left(a_{k-1},a_{k-1}^{\prime }\right)$ corresponds to the drifts $\delta_{k}\left(a_{k-1},a_{k-1}\right)$.

Using the stereographic projection of Listing’s sphere from the south pole −1 to the tangent plane $T_{1}S_{L}^{+}$, we can identify *P* with an 2n-gone on the tangent plane $T_{1}S_{L}^{+}$.

In the case of saccade, Listing’s curve is a segment $ab\subset S_{L}^{+}$. Hence all saccades of *n*-cycle are determined by the position of their initial and final points in Listing’s hemisphere, i.t. by 2n points $a_{k-1}^{\prime },a_{k},\;k=1,\cdots ,n$.

For example, a 3-cycle is characterised by the hexagon $a_{0}a_{0}^{\prime }a_{1}a_{1}^{\prime }a_{2}a_{2}^{\prime }a_{3},\;a_{0}=a_{3}=1$ and consists of 3 drifts and 3 MSs:

$$\delta_{1}=\delta \left(1,a_{0}^{\prime }\right),S_{1}=a_{0}^{\prime }a_{1},\delta_{2}=\delta \left(a_{1},a_{1}^{\prime }\right),S_{2}=a_{1}^{\prime }a_{2},\delta_{3}=\delta \left(a_{2},a_{2}^{\prime }\right),S_{3}=a_{2}^{\prime }1.$$

**Figure 11:**
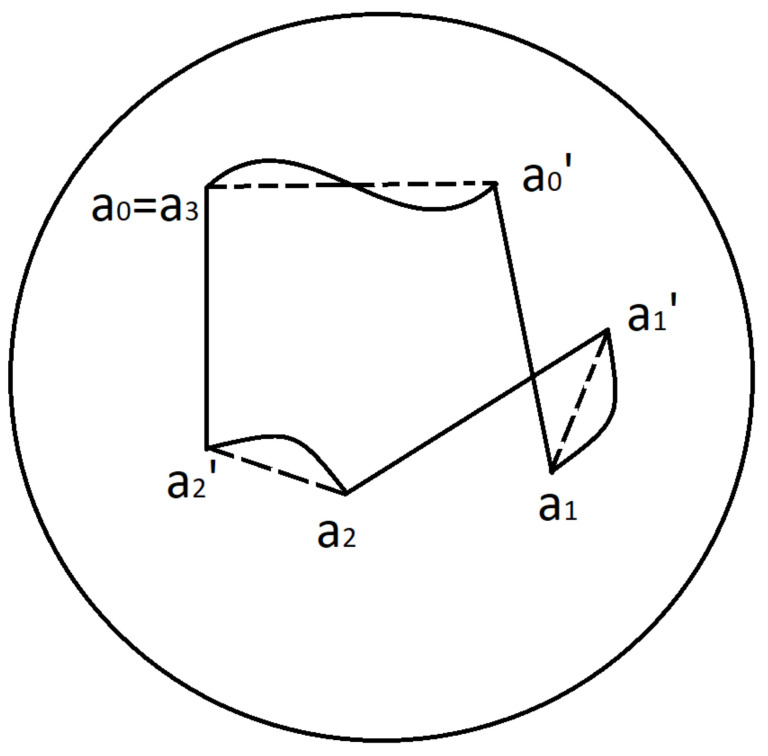
Hexagone.

An example of 3-cycle and associated hexagon is depicted in Figure 11.

We suppose that during *n*-cycle with a Listing’s curve a(t),t∈[0,T] the visual system perceives local information about the stimulus, more precisely, information about points *B* whose retinal image belong to the fovea. The information needed for such local pattern recognition during a FEM cycle consists of two parts:

(a) The dynamical information about Listing’s curve a(t),t∈[0,T], coded in oculomotor command signals. A copy of these signals (corollary discharge (CD)) is sent from the superior colliculus through the MD thalamus to the frontal cortex. It is responsible for visual stability, that is the compensation of the eye movements and perception of stable objects as stable.
(b) The visual information about characteristics of a neighborhood of points *B* of the stimulus which is primary encoded into the chain of photoreceptors along the closed retinal curve $\bar{B}\left(t\right)={\mathrm{Ad}}_{\bar{a}\left(t\right)}B\left(0\right)$, which represents the point *B* during FEM. Then this information is sent for decoding through LGN to the primary visual cortex and higher order visual structures. In particular, if $A\left(t\right)=\chi \left(a\left(t\right)\right)={\mathrm{Ad}}_{a(t)}\left(i\right)$ is the gaze curve with the initial direction to external point A∈RA(0)=Ri, the point of fixation *A* is represented by the retinal curve $\bar{A}\left(t\right)={\mathrm{Ad}}_{\bar{a}\left(t\right)}\left(i\right)$ with $\bar{A}\left(0\right)=-i$.

### 3.6. A Model of Fixation Eye Movements

At first, we consider a purely deterministic scheme for processing information encoded in CD and visual cortex.

Then we discuss the problem of extending this model to a stochastic model. We state our main assumptions. If the opposite is not stated, we assume that we are working in spatiotopic coordinates associated to $a_{0}=1$.

1. We assume that CD contains information about the eye position $a_{k-1}^{\prime },a_{k},\;k=1,\cdots ,n$ during the beginning and the end of the saccades $S_{k}$, (which is equivalent to information about the gaze positions) and about the corresponding time $T_{k}$.

2. We assume also that CD has information about Listing’s curve $\delta_{k}\left(t\right),\;t\in \left[T_{k-1},T_{k}\right]$ of the drift $\delta_{k+1}=\delta \left(a_{k},a_{k}^{\prime }\right)$ from the point $a_{k}$ to the point $a_{k}^{\prime }$. (This assumption is not realistic and later we will revise it.)

3. Let *B* be a point of the stable stimulus and $\bar{B}\left(0\right)$ its retina image at the time t=0. Then during the drift $\delta_{k+1}\left(t\right)=\delta \left(a_{k},a_{k}^{\prime }\right)$ the image of *B* is the retina curve ${\bar{B}}_{k+1}\left(t\right)={\mathrm{Ad}}_{{\bar{\delta }}_{k+1}\left(t\right)}\bar{B}$. We denote by $I_{k+1}^{B}\left(t\right)=I\left({\bar{B}}_{k+1}\left(t\right)\right)$ the characteristics of this image *B*, which is recorded in the activation of photoreceptors along the retinal curve $\bar{B}\left(t\right)$ during the drift $\delta_{k+1}$ and then in firing of visual neurons in V1 cortex and higher order visual subsystems. Note that the information about the external stable point *B* is encoded into the dependent on time vector–function $I_{k}^{A}\left(t\right)$. This is a manifestation of a phenomenon that E. Ahissar and A. Arieli [12] aptly named ‘figurating space by time’.

4. We assume that the (most) information about the drift $\delta_{k+1}\left(t\right)$, encoded in Listing’s curve $\delta_{k+1}\left(t\right)\subset S_{L}^{+}$ and about the characteristic functions $I_{k+1}^{B}\left(t\right)$, is encoded in the coordinate system, associated to the end point $a_{k}$ of the preceding saccade $S_{k}$. We remark that if $a_{k}=\cos\theta 1+\sin\theta p,\;p\in S_{L}^{1}$, then associated with $a_{k}$ coordinate system is obtained from the spatiotopic coordinates by the rotation along the axe *p* of the Listing plane Π(j,k) through the angle 2π. (These coordinates are the retinotopic coordinates at the time $T_{k}$).

5. Let *C* be another point of the stable stimulus with the retina image $\bar{C}\left(0\right)$ at t=0 and $I_{k+1}^{C}\left(t\right),\;t\in \left[T_{k},T_{k+1}\right]$ the characteristic function of the retina image

$$\bar{C}\left(t\right)={\mathrm{Ad}}_{{\bar{\delta }}_{k+1}}\left(t\right)\bar{C}\left(0\right)$$

of *C* during drift $\delta_{k+1}$. Then the visual system is able to calculate the visual distance between point B,C during drift $\delta_{k}$ as an appropriate distance between their characteristic functions $I_{k+1}^{B},\;I_{k+1}^{C}$.

6. We assume that the change of coordinates (remapping) appear during each saccade. So for example during 3-cycle, the system uses the coordinates associated to the following points of Listing’s hemisphere

$$a_{0}=1\;\;\left[0,T_{1}\right],\;a_{1}\;\;\left[T_{1},T_{2}\right],\;a_{2}\;\;\left[T_{2},T_{3}\right]$$

Here the interval $\left[T_{k},T_{k+1}\right]$ indicates the time of the drift $\delta_{k+1}$ when the coordinates $a_{k}$ is used.

7. In particular, this means that the information about the characteristic function $I_{k+1}^{B}\left(t\right)$ of the external point *B* along the retinal curves during the drift $\delta_{k+1}=\delta \left(a_{k},a_{k}^{\prime }\right)$ is encoded into the coordinates associated to the end point $a_{k}$ of the preceding saccade $S_{k}$ (which are the retinotopic coordinates at the time $T_{k}$).

To recalculate the characteristic function $I_{k}^{B}\left(t\right)$ in terms of the spatiotopic coordinates, associated to $a_{0}=1$, it is sufficient to know the point $a_{k}\in S_{L}^{+}$.

8. Following M. Zirnsak and T. Moore [46], we suppose that during the drift $\delta_{k+1}=\delta \left(a_{k},a_{k}^{\prime }\right)$, the visual system chooses an external saliency point *A* as the target for the next gaze position. More precisely, it fixes the retinal image $\bar{A}\in S_{E}^{2}$ of this point w.r.t. coordinates associated with $a_{k}$ (which are retinotopic coordinates at the moment $T_{k}$). After the next saccade $S_{k+1}=a_{k}^{\prime }a_{k+1}$ (at the moment $T_{k+1}$) the point $\bar{A}\in S_{E}^{2}$ will become the point *F* (the center of the fovea) and after the saccade the point *A* will be the target point of the gaze vector N=−F, A∈RN.

9. This allows to give an explanation of the presaccadic shift or receptive fields.

The above assumption means that before the time $T_{k+1}$ of the saccade $S_{k+1}$, the visual system knows the future gaze vector $e_{1}\left(T_{k}\right)=N=-F$ with respect to the coordinates, associated with $a_{k}$. Of course, this information may be obtained only due to collaboration of the visual system with the ocular motor system. At some moment $t_{shift}=T_{k+1}-\Delta <T_{k+1},\Delta \approx$ 100 ms these subsystems recalculate the characteristic functions $I_{k+1}^{B}\left(t\right)$ from the coordinates $a_{k}$ into the new coordinates, associated to the future gaze point $a_{k+1}$ and send this information to neurons of different visual systems.

This leads to the shift of receptive field, discovered in [45]. The information about the future characteristic functions will contains some mistakes since the real position of the eye at the moment $t_{shift}$ is different from the position $a_{k}$. It is observed as dislocation (compression) of the image in space and time [16,17,18,19]. After the saccade, this mistakes are corrected. One of the way to reduce such dislocation is to increase the frequency of microsaccades.

#### 3.6.1. Diffusion Maps and Stochastic Model of FEM

It seems that the assumption 1. that the CD contains information about the eye position at the beginning and the end of each saccade is rather reasonable. However, the assumption 2. must be clarified. Since the drift trajectory $\delta_{k+1}\left(t\right)$ (Listing curve) can be arbitrary, it is difficult to believe that the CD stores information about its shape even for a short time. It is naturally to assume that the drift is a random walk and the ocular motor system and CD store information about random trajectory of the drift. Similarly, the characteristic functions $I_{k+1}^{B}\left(t\right)$, which contain information about the stable stimulus *B*, recorded by photoreceptors during the drift $\delta_{k+1}$ becomes a random function.

#### 3.6.2. Diffusion Map by R.R. Coifman and S. Lafon

We shortly recall the basis ideas of the diffusion maps (or diffusion geometry) by R.R. Coifman and S. Lafon [34,35], which we will need.

The diffusion geometry on a (compact oriented) manifold *M* with a volume form dvol such that $\int_{M}d\mathrm{vol}=1$ is determined by a **kernel** k(x,y) i.e., a non negative and symmetric ((k(x,y)=k(y,x)≥0) function on M×M. An example of the kernel is the Gauss kernel $k_{\epsilon }\left(x,y\right)=\exp\frac{-||x-{y||}^{2}}{\epsilon },\;\epsilon >0$, of the Euclidean space or the heat kernel of a Riemannian manifold. The normalization of the kernel gives the **transition Markov kernel**

$$p\left(x,y\right):=\frac{k(x,y)}{d(x)}:=\frac{k(x,y)}{\int_{M}k\left(x,y\right){\mathrm{vol}}_{\left(y\right)}}.$$

which defines a random walk on *M*. The value p(x,y) is considered as the probability to jump in one step from the point *x* to the point *y*.

The associated diffusion operator *P* on the space of function is defined by

$$\left(Pf\right)\left(x\right)=\int_{M}p\left(x,y\right)f\left(y\right){\mathrm{vol}}_{\left(y\right)}.$$

Then the probability density to move form *x* to *y* in T∈N steps is described by the kernel $p^{T}\left(x,y\right)$ associated to the T∈N power $P^{T}$ of the operator *P* such that

$$\left(P^{T}f\right)\left(x\right)=\int_{M}p^{T}\left(x,y\right)f\left(y\right){\mathrm{vol}}_{\left(y\right)}.$$

It can be defined for any T∈R in terms of the eigenvectors and eigenfunction of the operator *P* [34]. So any point x∈M determines a family of the **bump functions** $p_{x}^{T}\left(u\right):=p^{T}\left(x,u\right)$ on *M*, which characterize the local structure of a small neighborhood of *x*. We call $p_{x}^{T}\left(u\right)$ the **trajectory of random walk** (or **random trajectory**) started from *x* during time interval [0,T].

R.R. Coifman and S. Lafon [34] define the **diffusion distance** between points x,y∈M as the $L_{2}$-distance between the bump functions (or random trajectories) $p_{x}^{T}\left(u\right)$ and $p_{y}^{T}\left(u\right)$, started form these points:

$${\left(D_{T}\left(x,y\right)\right)}^{2}=\left|\right|p_{x}^{T}-p_{y}^{T}{\left|\right|}^{2}:=\int_{M}{\left[p_{x}^{T}\left(u\right)-p_{y}^{T}\left(u\right)\right]}^{2}d{\mathrm{vol}}_{\left(u\right)}.$$

Let $\lambda_{0}=0<\lambda_{1}\leq \lambda_{2}\leq \lambda_{3}\cdots$ be eigenvalues of the diffusion operator *P* and $\psi_{0}=1,\psi_{1},\psi_{2},\cdots$ associated eigenfunctions. Then for sufficiently big number *m*, the diffusion distance $D_{T}\left(x,y\right)$ is approximated by the function $D_{m,T}\left(x,y\right)$ given by

$$D_{m,T}^{2}\left(x,y\right)=\sum_{k=1}^{m}(\lambda_{k}^{2T}{\left(\psi_{k}\left(x\right)-\psi_{k}\left(y\right)\right)}^{2}.$$

In other worlds, the map (called the **diffusion map**)

$$\Phi_{T}:M\to R^{m},\;x\mapsto \Phi_{T}={\left(\lambda_{1}^{t}\psi_{1}\left(x\right),\lambda_{2}^{t}\psi_{2}\left(x\right),\cdots ,\lambda_{m}^{t}\psi_{m}\left(x\right)\right)}^{t}$$

is closed to the isometric map of the manifold *M* with the diffusion metric $D_{T}$ to the Euclidean space $R^{m}$. If the manifold *M* is approximated by a finite systems of points $X=(x_{1},x_{2},\cdots ,x_{N})$, the diffusion map gives a dimensional reduction of the system *X*.

#### 3.6.3. Remarks on Stochastic Description of Drift as Random Walk and Possible Application of Diffusion Distance

The idea that FEMs is a stochastic process and may be described as a random walk has a long history, [29,30,31,32,33,42].

1. We assume that the drift is a random walk on the Listing hemisphere $S_{L}^{+}$ defined by some kernel. The question is to chose an appropriate kernel. The first guess is to assume that it is the heat kernel of the round (hemi)sphere. The short-time asymptotic of the heat kernel of the round sphere is known, see [47]. The functional structure of the retina which records light information, is very important for choosing the kernel. Inhomogeneity of the retina shows that the first guess is not very reasonable. It seems that the more natural assumption is that the system uses the heat kernel for the metric on Listing hemisphere, which corresponds to the physiological metric of the retina. Recall that it is the pull back of the physical metric of the V1 cortex with respect to the retinotopic mapping.

2. We assume that the drift is a random walk in Listing’s hemisphere, defined by some kernel. Then by the drift trajectory $\delta_{k+1}\left(t\right)$ from the point $a_{k}$ we may understand the random trajectory on $S_{L}^{+}$ (or the bump function) $p_{a_{k}}\left(a\right):=p_{a_{k}}^{\Delta T}\left(a\right)$ during the time interval $\Delta T=[T_{k},T_{k+1}]$. It has no fixed end point but it allows to calculate the probability that the end point belongs to any neighbourhood of the point $a_{k}^{\prime }$. The situation is similar to Feynman’s path integral formulation of quantum mechanics. Moreover, if by a point we will understand not a mathematical point but a small domain, e.g., the domain which corresponds to the receptive field of a visual neuron in V1 cortex or the composite receptive field of a V1 column (which is 2–4 time larger) [37], then we may speak about random drift $\delta a_{k},a_{k+1}$ from the point $a_{k}$ to the point $a_{k+1}$ with the bump function $p_{a_{k},a_{k+1}}^{\Delta T}\left(a\right)$ (“the random trajectory”). Roughly speaking, this function gives the probability that the random drift from the point $a_{k}$ to the point $a_{k+1}$ after ΔT steps comes to the point $a\in S_{L}^{+}$.

3. Due to diffeomorphism defined by the Hopf map $\chi :S_{L}^{+}\to {\tilde{S}}_{E}^{2}$, we may identify the random walk in $S_{L}^{+}$ with the random walk on the eye sphere ${\tilde{S}}_{E}^{2}$. A drift $\delta_{k+1}\left(t\right)=\delta \left(a_{k},a_{k+1}\right)$ in $S_{L}^{+}$ induces the “drift” of a point $B\in {\tilde{S}}_{E}^{2}$ given by

$$B\left(t\right):={\mathrm{Ad}}_{{\bar{\delta }}_{k+1}\left(t\right)}B.$$

Let *A* be the fixation point of the gaze at the initial moment t=0, such that its retina image is −i. Then the retina image of the point *A* during the drift $\delta_{k+1}\left(t\right)$ is the curve $A\left(t\right)={\mathrm{Ad}}_{{\bar{\delta }}_{k+1}}\left(-i\right)$. More generally, if B(0) is the retina image at t=0 of any other point *B* of the stimulus, then the retina image during the drift $\delta_{k+1}$ is

$$B\left(t\right):={\mathrm{Ad}}_{{\bar{\delta }}_{k+1}\left(t\right)}B\left(0\right).$$

In the stochastic case, the drift ${\bar{\delta }}_{k+1}\left(t\right)$ is characterized by the random trajectory $p_{a_{k}}^{\Delta T}\left(a\right)$, and associated “drift” of points in ${\tilde{S}}_{E}^{2}$ by the random trajectory

$$p_{B_{k}}^{\Delta T}\left(x\right):=p_{a_{k}}^{\Delta T}\left(sx\right)$$

where $B_{k}={\mathrm{Ad}}_{a_{k}}B\left(0\right)$ and $s=\chi^{-1}$ is Listing’s section. Note that the right hand side does not depend on the point B(0).

We conjecture that the ocular motor control system detects information about random trajectories in $S_{L}^{+}$ and $S_{E}^{2}$ and the corollary discharge get a copy of this information.

It seems that the proposed explanation for shifting receptive fields may be generalized to the stochastic case.

4. Let *B* be a stable stimulus and $B_{0}$ its retina image at t=0 and $B_{k}:={\mathrm{Ad}}_{{\bar{a}}_{k}}B_{0}$ the retina image at the time $T_{k}$. Denote by $I_{k}^{B}\left(t\right)=I\left(B_{k}\left(t\right)\right)$ the characteristic function, which describes the visual information about a stable stimulus point *B* with the retina image $B_{k}\left(t\right)$ during the drift $B_{k}\left(t\right),t\in \left[T_{k},T_{k+1}\right]$. If the drift is considered as a random walk, the information about the drift curve $B_{k}\left(t\right)\subset S_{E}^{2}$ is encoded in the function $p_{a_{k}}^{\Delta T}\left(sx\right)$ and the characteristic function $I_{k}^{B}\left(t\right)$ becomes a random function and is described by the bump function $p_{I_{k}^{B}}^{\Delta T}\left(x\right)$ on $S_{E}^{2}$. We suppose that the visual system calculates the visual distance between external points B,C as the diffusion distance between the associated bump functions.

5. We also conjecture that like in deterministic case, the information about the random trajectory of the drift $\delta_{k+1}$ encoded in CD and the information about characteristic bump function, encoded in different structures of the visual cortex are sufficient for stabilization of visual perception. The problem reduces to recalculation of all information in spatiotopic coordinates, associated with the point a=1.

## Figures and Tables

**Table 1:** Characteristics of fixation eye movements (Adapted from [44]) with refined data from [23,43] and Wikipedia.

	Amplitude	Duration	Frequency	Speed
Tremor	11-60 arcsec	-	50–100 Hz	Max 20 arcmin/s
Drift	1.5–4 arcmin	0.2–0.8 s	95–97% of time	50 arcmin/s
Micsac	1–30 arcmin	0.01–0.02 s	0.1–5 Hz	40–220 deg/s
